# Simultaneous combined anterior and posterior approach for en bloc resection of sciatic notch sarcomas

**DOI:** 10.1186/s12893-019-0488-6

**Published:** 2019-02-20

**Authors:** Elodie Gaignard, Dimitri Tzanis, Toufik Bouhadiba, David C. Kieser, Fabien Robin, Damien Bergeat, Bernard Meunier, Sylvie Bonvalot

**Affiliations:** 1 Service de chirurgie hépatobiliaire et digestive, CHU Rennes, CHU Pontchaillou, 2 rue Henri le Guilloux, 35033 Cedex 9, Rennes, France; 2 Université de Rennes 1, Rennes, France; 3 Department of Surgery, Institut Curie, PSL Research University, Paris, France; 4 Department of Orthopaedic Surgery and Musculoskeletal Medicine, Canterbury District Health Board, Christchurch, New Zealand

**Keywords:** Sarcoma, Sciatic notch, Surgery, Anterior approach, Posterior approach, Sarcome, échancrure sciatique, Chirurgie, Approche antérieure, Approche postérieure

## Abstract

**Background:**

Monobloc resection of soft tissue sarcomas (STSs) has a major impact on overall survival and local recurrence. Anatomical boundaries, such as the sciatic notch, increase the risk of fragmentation of the lesion. To date there are few papers describing the optimal surgical technique to remove such STSs. The objective of this study is to describe a simultaneous anterior and posterior approach for resection of sciatic notch dumbbell tumours.

**Case presentation:**

We present the surgical management of two patients diagnosed with well-differentiated liposarcomas of the sciatic notch with a retroperitoneal and gluteal extension in the two cases. Pre-operative diagnosis was made with a percutaneous biopsy including molecular analysis which demonstrated MDM2 amplification. We describe a simultaneous anterior and posterior approach, including the ligation of the posterior trunk of the internal iliac artery, to reduce intra-operative blood loss and devascularise the tumour. The anterior approach allows the evaluation of the tumour’s retroperitoneal extension, release from its pelvic attachments and control of the surrounding neurovascular structures. During the posterior approach, bleeding is reduced by the devascularisation of the gluteal musculature achieved with internal iliac artery ligation. Clear margins were achieved in both cases. No vascular, skeletal or soft tissue reconstructions were required.

**Conclusions:**

Simultaneous combined anterior and posterior approaches to remove a malignant sciatic notch tumour optimises the chance of complete en bloc resection. This surgical strategy allows oncologic en bloc resection with minimal blood loss.

## Background

Soft tissue sarcomas (STSs) are a heterogeneous group of malignant neoplasms arising from mesenchymal tissues and are named after the site or type of tissues affected. The incidence of STS is estimated at 30 cases/million population per year [1, 2].

Due to the rarity of these tumours, histological diversity and varied presentations, the global management of these tumours remains poor [3]. To combat this, the NETSARC group was established in 2009, by the French National Cancer Institute. Its aim was to improve the health care of patients with STSs. The main objective of this professional network is to develop guidelines to improve health care and global survival of patients with a sarcoma. However, deviations from guidelines and inadequate management of these patients remain common and thus considerably impact on patients’ survival [4, 5]. To limit this, working groups of experts have defined “reference centres” to ensure appropriate management of these rare conditions. An institution is considered as a sarcoma centre if it treats at least 100 STSs and 50 bone sarcomas annually [6]. In addition, a sarcoma referral centre must include a radiologist, pathologist, surgeon and oncologist, with decisions made as a multidisciplinary team (MDT), and according to the European Society of Medical Oncology (ESMO) guidelines [7]. This approach has been supported by several studies reporting that the expertise of the treating centre is one of the most important factors determining survival and recurrence in STSs [8].

While multiple adjunctive therapies are used in STSs, surgical resection by a sarcoma surgeon remains the standard of care for these tumours [9, 10]. This surgery typically involves an en bloc resection with negative margins (R0). Fortuitously, most STSs affect the extremities and such an approach can often be achieved. However, some STSs affect the retroperitoneum and sciatic notch [11]. In these cases, the complex regional anatomy and rare incidence of these so called “dumbbell-shaped” STSs present a significant surgical challenge with a high risk of bleeding and tumour fragmentation.

Descriptions of such tumours and the surgical techniques employed to resect these lesions are limited. Most reports concerning tumours involving the sciatic notch are case reports [12–14]. Our aim is to describe our surgical technique to resect dumbbell-shaped STSs of the sciatic notch.

## Case presentation

Institutional review board and patient consents were obtained to present these cases.

Two patients with a dumbbell-shaped STS of the sciatic notch presented to our sarcoma centre. Patient 1 presented with asymmetric buttocks causing embarrassment when sitting. Patient 2 presented with constipation. Both patients’ clinical examination revealed a deep, non-mobile mass in the buttock, but normal neurologic and functional examinations.

**Fig. 1:**
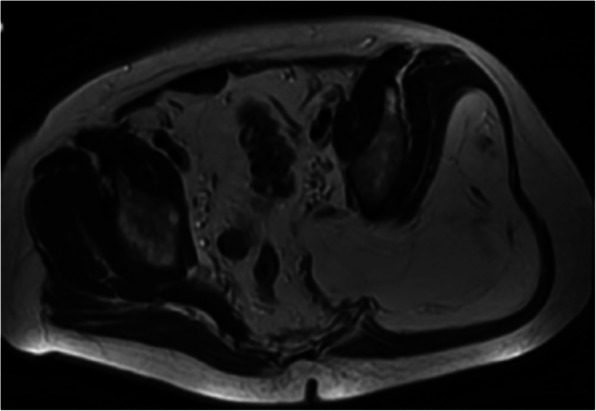
Patient 1: MRI of the liposarcoma within the sciatic notch

**Fig. 2:**
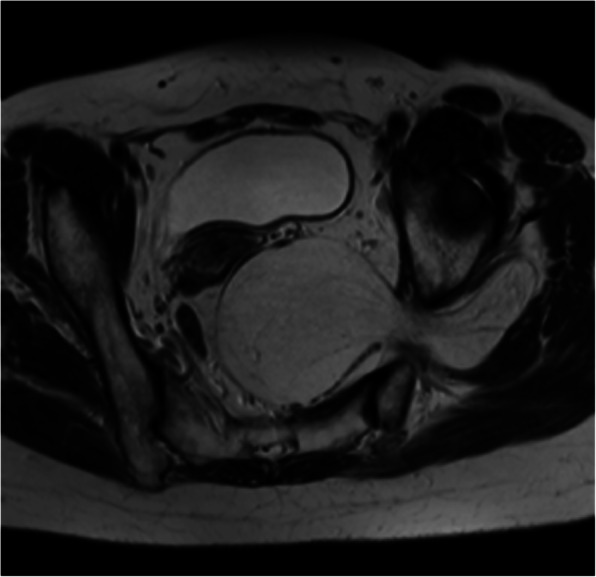
Patient 2: Note the tumours retroperitoneal as well as large gluteal extension

In both patients, an MRI scan revealed a large lipomatous lesion crossing the sciatic notch (Figs. 1 and 2). In patient 1 the largest extension was in the buttock with a large infiltration of the gluteus maximus muscle, but with no extension into either the gluteus medius or minimus muscles. Conversely, in patient 2, the maximum extent was the retro-peritoneal space. In both, the tumour was in contact with the iliac vessels, ureter and sciatic nerve.

In both patients, multiple core biopsies with 14G needles were performed under ultrasound via a posterior approach by an interventional radiologist. Samples were analysed by a pathologist from the Réseau de Référence en Pathologie des Sarcomes des tissus mous et des viscères group, and revealed a well-differentiated liposarcoma with amplification of MDM2 on the fluorescent in situ hybridisation (FISH). After the MDT, the decision was made for an en bloc surgical resection.

## Surgical technique

The main surgical objective of STS resections is to achieve complete resection with clear margins. Tumour breach or piece-meal resection exposes patients to local dissemination and therefore recurrence. In order to achieve an en bloc resection and limit injury to the uninvolved surrounding structures, we used a simultaneous combined anterior and posterior approach with the patient in a lateral decubitus position.

**Fig. 3:**
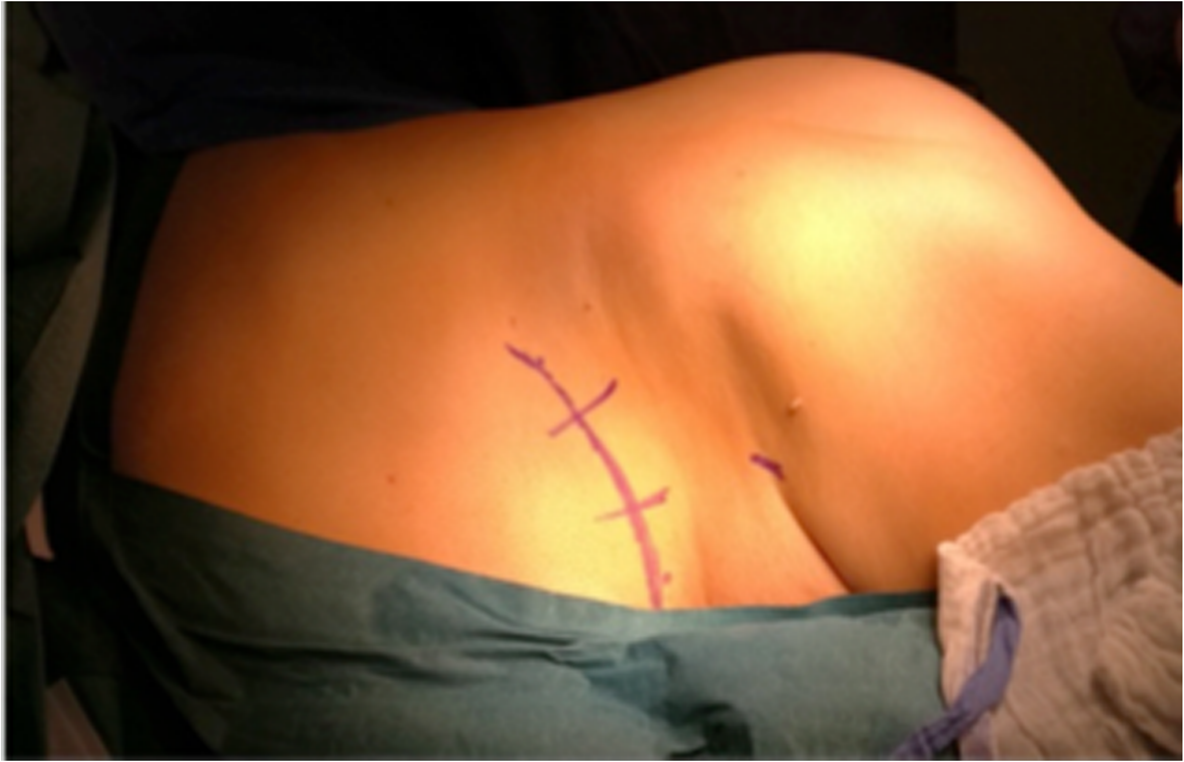
Anterior approach

We started with an anterior approach to release the tumour from its pelvic attachments. This involves a curvi-linear para-rectal incision parallel to the inguinal ligament and retroperitoneal approach to the tumour (Fig. 3). In the retroperitoneal space, we release the iliac vessels, ureter and obturator nerve. This approach exposes the internal iliac vessels. The posterior trunk of the internal iliac artery gives rise to both the superior gluteal and lateral sacral arteries, which are significant contributors to the blood supply to the gluteal region. Because of the large tumour extensions into the gluteal regions in these cases and necessity for an en bloc resection, ligation of the posterior trunk was performed to limit perioperative haemorrhage during the posterior resection and significantly reduce the tumoural blood supply.

After ligature of the vessels, dissection towards the sacrum exposed the greater sciatic notch. In patient 2, it was necessary to incise the sacrospinous and sacrotuberous ligaments to enlarge the sciatic foramen to achieve delivery of the tumour through the notch [15]. Osteotomies of the sciatic notch margins were not required in these cases.

**Fig. 4:**
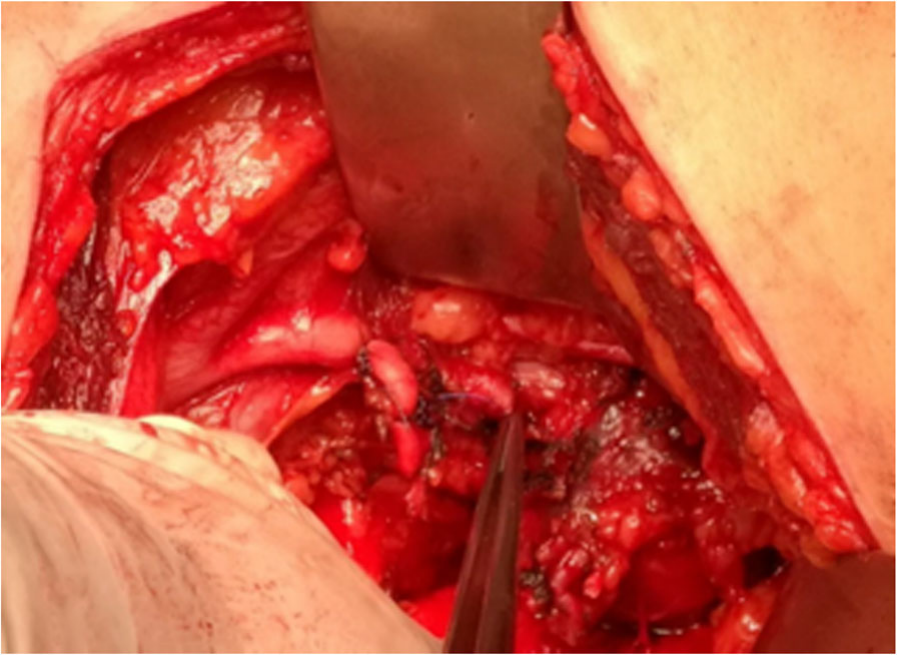
Ligature of the left hypogastric artery. Obturator nerve and sacral root S1 are visible

The tumour was then freed circumferentially, completely separating it from its pelvic attachments, including the hypogastric vessels, ureter, obturator nerve, sacral nerve roots, bladder and rectum (Fig. 4).

**Fig. 5:**
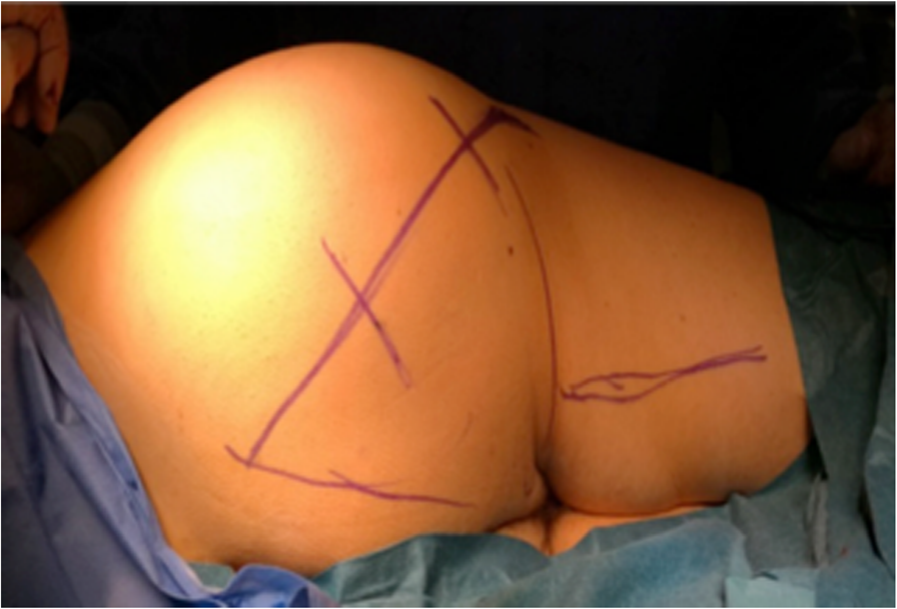
Skin markings for the oblique posterior gluteal incision. The region of the sciatic nerve is also marked

With the patient in the same position we then performed the posterior approach to deliver the tumour. This approach was performed through an oblique gluteal incision extending from the posterior-superior iliac spine (PSIS) to the greater trochanter (Fig. 5).

**Fig. 6:**
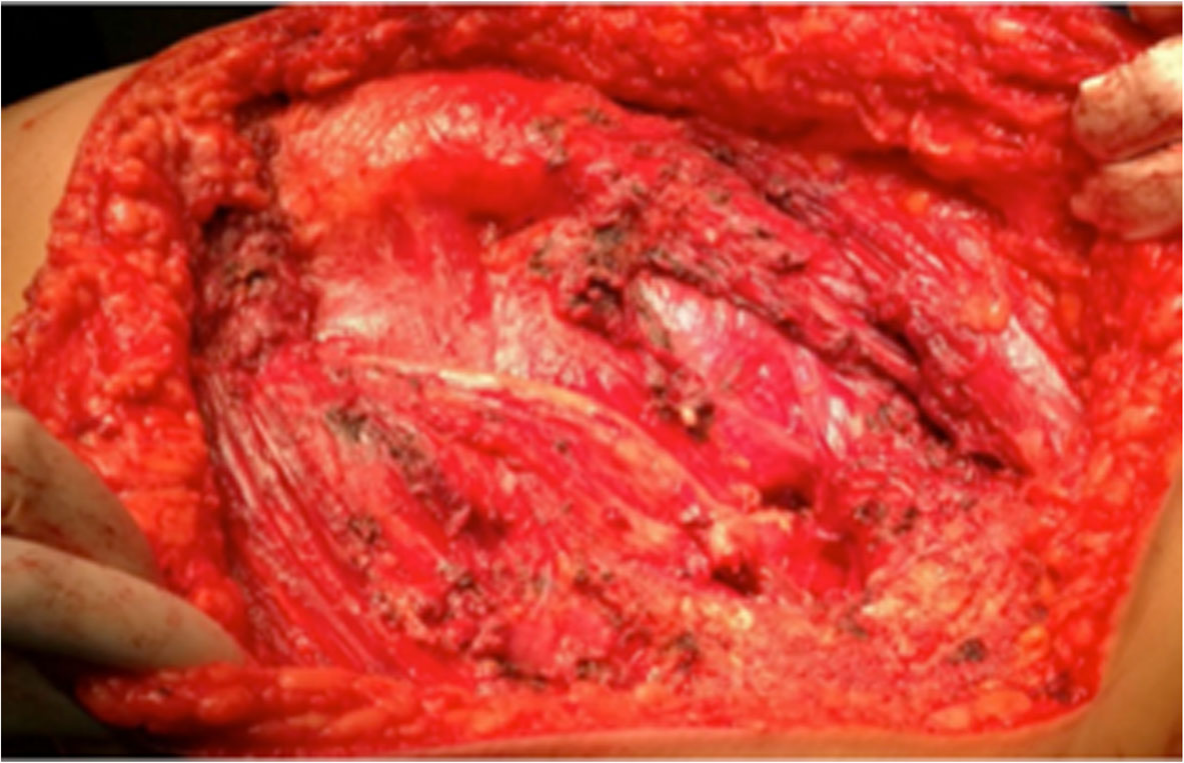
Exposition of the hip joint, sciatic nerve and gluteus minimus after excision of the tumour

A subcutaneous plane was then developed to expose the gluteus maximus. After exposing the medial and lateral borders of the muscle, it was released from its insertion into the linea aspera and fascia lata. The gluteus maximus and its contained tumour were then elevated superiorly. The hip abductors, hip joint, hamstrings, posterior cutaneous nerve of the thigh and sciatic nerve were protected (Fig. 6).

After initial elevation of the inferior portions of the muscle, the medial origin of the gluteus maximus was identified along the para-sacral region from the coccyx to posterior-inferior sacroiliac joint (PISIJ). This was elevated with caution to avoid damage to the superior and inferior gluteal arteries. Understanding that the superior gluteal artery is typically located within 5mm of the PISIJ and the inferior gluteal artery is located in the midpoint between the PISIJ and sacrococcygeal joint, aided their identification [16]. The resected specimen was then freed circumferentially and working through both incisions is delivered through the posterior incision. The wounds were then closed in a standard fashion.

Post-operatively, both patients had an unremarkable recovery with no functional sequelae. Estimated blood loss during surgery were less than 200ml and mean operation time in the 2 cases was 210 min.

**Fig. 7:**
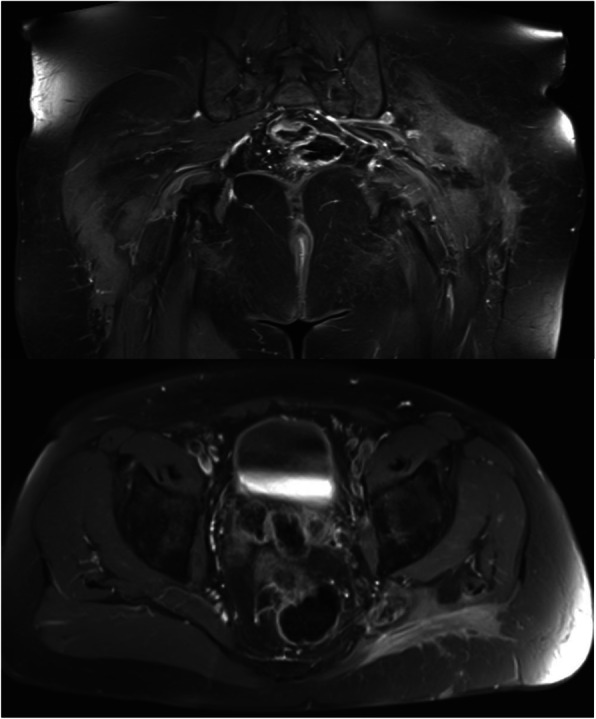
MRI follow up 12 months after surgical resection

They were discharged 9 and 10 days post-operatively respectively. The definitive diagnosis confirmed that both were well-differentiated liposarcomas, Fédération Nationale des Centres de Lutte Contre le Cancer (FNCLCC) grade 1. In these two cases, surgical margins were marginal only around the neurovascular structures of the sciatic notch. According to the recommendations of the European Society of Medical Oncology, postoperative radiotherapy is not required for well-differentiated liposarcomas even if excision is marginal. In both patients at 12 months follow up, there was no evidence of recurrence or metastatic disease and their neurological functions were normal (Fig. 7).

## Discussion

Soft tissues sarcomas of the sciatic notch pose a challenging surgical problem due to their rareness and complex regional anatomy, with a balance needed between adequate oncologic resection margins and preservation of function [17].

In order to optimise surgical resection, but also limit inadvertent injury to uninvolved tissues, we performed a simultaneous dual anterior and posterior approach. During the anterior approach we ligated the posterior trunk of the internal iliac artery to aid exposure of the sciatic notch, reduce the tumour’s blood supply and control intra-operative bleeding. We feel that ligation of the posterior branch of the internal iliac artery allows control of the superior gluteal artery, which predominantly supplies the gluteus maximus muscle, which in our case was completely removed. In general, the total excision of the gluteus maximus muscle has little functional impact on a normal gait and stable pelvis on standing, as was the case in our two patients.

Although not necessary in these cases, ligation of the internal iliac artery proper may be necessary. The ligation of the internal iliac artery exposes the patient to a rectal ischemia or buttock claudication. But, in the case of sciatic notch sarcomas, this complication remains very rare because of the large anastomotic connections with the contra-lateral internal iliac artery and branches. A preoperative computed-tomography with 3-dimentional reconstruction can be utilised to determine the pelvic arterial anastomotic networks. So, with adequate planning, we feel that this technique has no significant effect on the pelvic structures. Similarly, cross-sectional imaging can help determine whether sciatic notch osteotomies are required or whether the sciatic nerve will need to be sacrificed during the resection. This aids in education of the patient prior to the procedure and allows them to understand the risks of sciatic nerve palsy, osteomyelitis, hip or sacro-iliac disruption.

In our cases, we had to accept a marginal excision around the neurovascular structures of the sciatic notch, especially the gluteal and sciatic nerves in order to preserve post-operative function. En bloc resection is necessary to avoid peritoneal seeding, but marginal excision is accepted for well differentiated LPS, especially in such locations [18]. Our policy for well-differentiated LPS is to spare adjacent critical structures even if it leads to a marginal excision. In 2017, Bonvalot and colleagues showed that a minimum surgical margin of 2mm was enough to obtain local control in these tumours [19]. We feel this margin is often attainable through the sciatic notch as the critical structures are usually pushed to the peripheries of the notch rather than encased by the lesion. Moreover, according to the recommendations of the European Society of Medical Oncology, postoperative radiotherapy is not required for well-differentiated liposarcomas even if excision is marginal. However, in the case of fragmentation of the tumor, postoperative radiotherapy should be considered for local control.

In our second case the circumference of the sciatic notch was insufficient to deliver the retroperitoneal extension of the tumour through the sciatic notch, thus, release of the sacrospinous and sacrotuberous ligaments was necessary. Although not required in our cases, marginal osteotomies may be used to enlarge the sciatic foramen. Li and colleagues (2017) performed a C-shaped osteotomy during the posterior approach with a piezoelectric bone cutter preventing damage to the hip and sacroiliac joint [15]. We support this technique as it protects the soft tissues while the bone is resected.

Interestingly, Spinner and colleagues (2006) reported fives cases of combined anterior and posterior approaches to remove benign tumours of the sciatic notch [20]. In this study, the authors suggested that it was not necessary to expand the sciatic notch. However, in the case of malignant tumours, such as in our case, debulking the retroperitoneal portion of the tumour risks tumour contamination and therefore we advocate an expansion osteotomy if needed to deliver the tumour. Thus, surgeons performing these procedures should be prepared to undertake expansion osteotomies, because although the likelihood of requiring a sciatic notch osteotomy can be pre-operatively estimated with cross-sectional MRI imaging, the stiffness of the tissues and tumour adherence to the sciatic notch sidewalls can only be appreciated intra-operatively [21].

## Conclusions

A simultaneous combined anterior-posterior approach to remove STSs of the sciatic notch offers direct access to the tumour mass with circumferential release and the ability to deliver the tumour safely through the sciatic notch. When the tumour has a large posterior extension involving the gluteus maximus, ligation of the posterior branch of the internal iliac artery can be used to reduce intra-operative blood loss during the posterior approach. Complete excision of the gluteus maximus ensures adequate margins and appears to have little functional effect.

## Abbreviations

ESMO: European Society of Medical Oncology
MDT: MultiDisciplinary Team
RRePS: Réseau de Référence en Pathologie des Sarcomes des tissus mous et des viscères
STS: Soft tissue sarcoma
